# Mercy Hospital Clinic:—Cardiac Disease with Œdema of the Lungs, Etc.—Inflammation of Knee-Joint

**Published:** 1867-02

**Authors:** N. S. Davis


MERCY HOSPITAL CLINIC:—CARDIAC DISEASE WITH ŒDEMA OF THE LUNG, ETC.—INFLAMMATION OF KNEE-JOINT.—CLINIC BY PROF. N. S. DAVIS.—*Ward No. 9.* The attention of the class was first called to Mr. B., a German, aged about 24 years. The lecturer reminded the members of the class that they had already once carefully examined the same patient, immediately after his admission to the hospital, eight days previously. At that time, the patient was sitting up in bed, the knees drawn up and the arms resting on them for the purpose of supporting the body in a position a little inclined forward, with extreme dyspnœa. The face was somewhat bloated; expression dull, with constant drowsiness; and the lips, nose, and hands were of a livid or dark purplish color, and colder than natural. The pulse was quick, small, and irregular, and the heart's action irregular, but tumultuous and violent. The tongue was clean; the mouth moist; bowels regular; urine natural though scanty; but loss of appetite and great sense of weakness. Percussion revealed entire dullness over the lower part of the right side. Auscultation afforded no respiratory sound over the whole left side, and only a coarse rhonchus over the upper and anterior part of the right. From the extreme difficulty of breathing and the irregular and violent action of the heart, it was almost impossible to appreciate the cardiac sounds. All that could be learned concerning the history of the case was, that he had been troubled with frequent paroxysms of dyspnœa, called asthma, for two or three years, and a constantly increasing shortness of breath on attempting to take exercise.

In analyzing the symptoms and physical signs, at that time, with a view to diagnosis, it was stated that the dyspnœa, the dullness on percussion, and the absence of respiratory murmur, all indicated greatly increased density of the lungs and a corresponding diminution of capacity for air. And it was stated that such increased density might depend on three different pathological conditions, namely, pneumonic hepatization, compression from pleuritic effusion, and œdema or serous infiltration of the pulmonary tissue. That it was not the first, in the case before us, was evident from the absence of fever, of pneumonic expectoration, and of those rhonchi which are always present in some degree in hepatization, and from the attitude of the patient. We never see a patient with hepatization of the lung sufficiently extensive to render a whole side dull on percussion, and face livid from decarbonization of the blood, habitually seeking to sit upright in bed with the arms resting on the knees, as was the case with this patient. That it was not pleuritic effusion, was equally evident, from the absence of enlargement of the side most affected, the absence of any bulging of the intercostal spaces, and the absence of displacement of the heart; for if the left side of the chest was filled with serous effusion sufficient to suppress the respiratory murmur and render the whole side dull, it would necessarily enlarge the side and crowd the heart towards the sternum.

The class will remember that by this negative process of investigation, as well as by the positive character of the existing symptoms, it was announced as the opinion of the lecturer, that the patient was laboring under extensive œdema of the left lung, with partial œdema of the lower lobe of the right. Whether this state of the lungs had resulted from primary valvular disease of the heart, more especially of the mitral valve, which would obstruct the return of blood from the lungs, or from such a degree of constriction of the bronchial tubes as had prevented sufficient ingress of air to oxygenate and decarbonize the blood, could not be positively determined at that time for reasons already given. The immediate indications for treatment, however, were obvious. The patient was suffering the direct effects of defective aëration of the blood, while the heart was violently sending the blood to the already engorged and infiltrated lungs faster than it is capable of passing through the pulmonary capillaries. Hence, to increase the oxygenation of the blood, and moderate the violent and unsteady action of the heart, were indications of pressing importance. To accomplish the first, the patient was ordered to have the following:—

℞. Chlorate of Potass.,-------------------- ʒij.

Pulv. G. Arabic,---------------------- ʒiij.

Mix and dissolve in an ordinary tumbler full of cold water, and take a *tablespoonful* every two hours, until the blueness of the lips, nose, and hands was diminished, and then lengthen the intervals to once in four hours. To meet the second indication, three drops of the saturated tincture of veratrum viride were directed to be taken between each of the doses of the chlorate of potassa, and the quantity increased or diminished as should be found necessary to keep the action of the heart more quiet and uniform. This treatment was continued for three days, with a slow but decided improvement in the condition of the patient. The heart's action became more steady; the pulse more regular; the lips, nose, and hands more natural color; and the dyspnœa less. Six-grain doses of iodide of potassa were then given in place of the chlorate, every four hours, and six drops of tinct. gelseminum alternated with it in place of the veratrum viride. This has constituted the treatment until the present time. The patient is now resting in the recumbent position; pulse nearly regular; skin natural in temperature, and much improved in color; but the respiration is still very short and inflation of the lungs very imperfect, with frequent suffocating cough; scanty and tenacious expectoration, and great sense of weariness, yet unable to obtain refreshing sleep.

The patient being quiet and resting in an easy position, each member of the class was required again to listen to the cardiac and respiratory sounds. The heart now acting more slowly and regularly than at the first examination, it was easy to distinguish a rough sound continuous after the first sound of the heart and coïncident with the diastole of the ventricles. It was most audible about one inch below the nipple, nearly over the apex of the heart, thus indicating disease of the mitral valve sufficient to partially obstruct the left auriculo-ventricular opening. On extending the examination to the lungs, it was found that the right lung expanded much more freely than at the time the patient was admitted, although there was still some dullness and sub-mucous rhonchus over the lower and posterior part of that side. The upper lobe of the left lung had become partially permeable, as indicated by some rhonchus, but the dullness was still strongly marked. And the sudden, short expiratory act showed that far less than the natural quantity of air entered the lungs by inspiration. The present examination confirmed the previous diagnosis, so far as related to the pulmonary œdema, and rendered it quite certain that it was complicated with organic valvular disease of the heart. The existence of the latter, rendered the prognosis unfavorable, so far as related to full recovery. And yet, the continuance of judicious treatment may so far improve the condition of the patient as to enable him to take some moderate degree of exercise. The objects to be accomplished by treatment are still the same as were pointed out at the previous clinic, namely, to control the excessive action of the heart; to hasten the reäbsorption of the serous effusion or infiltration into the pulmonary tissue; and to promote the rest of the patient. To accomplish these purposes, the patient was directed a simple but nutritious diet; six drops of the saturated tincture of gelsemium half an hour after each meal, and a teaspoonful of the following emulsion every four hours:—

℞. Chloroform,---------------------------------- ʒiij.

Fl. Ext. Cannabis Ind.,------------------------ ʒiij.

Iodide Potassa,-------------------------------- ʒiij.

Pulv. G. Arabic,-------------------

White Sugar, ----------------------- } āā ʒiv.

Rub together, and add,
Mint Water,------------------------------------ ℥iij.

Mix.

Case II. The second case presented to the class during the clinic hour, was one in which there had been sub-acute inflammation of the synovial membrane and cartilages of the knee-joint; but which had nearly disappeared under the influence of entire rest and a succession of blisters over different parts of the joint. Of this, our space will not admit even a brief report.

